# Impact of diabetes mellitus on long-term clinical and graft outcomes after off-pump coronary artery bypass grafting with pure bilateral skeletonized internal thoracic artery grafts

**DOI:** 10.1186/s12933-022-01687-2

**Published:** 2022-11-15

**Authors:** Ilkun Park, Kuk Bin Choi, Joong Hyun Ahn, Wook Sung Kim, Young Tak Lee, Dong Seop Jeong

**Affiliations:** 1 Department of Thoracic and Cardiovascular Surgery, Samsung Medical Center, Sungkyunkwan University School of Medicine, 81 Irwon-Ro, Gangnam-Gu, Seoul, 06351 Republic of Korea; 2 Department of Cardiovascular Surgery, Mayo Clinic, Rochester, Minnesota USA; 3 Biostatistics and Clinical Epidemiology Center, Samsung Medical Center, Sungkyunkwan University School of Medicine, Seoul, Republic of Korea; 4 Department of Thoracic and Cardiovascular Surgery, Incheon Sejong Hospital, Incheon, Gyeonggi-Do Republic of Korea

**Keywords:** Diabetes mellitus, Coronary artery bypass graft, Internal thoracic artery, Graft failure, Wound infection

## Abstract

**Background:**

The effect of diabetes mellitus (DM) on the long-term outcomes of coronary artery bypass graft (CABG) remained debatable and various strategies exist for CABG; hence, clarifying the effects of DM on CABG outcomes is difficult. The current study aimed to evaluate the effect of DM on clinical and graft-related outcomes after CABG with bilateral internal thoracic artery (BITA) grafts.

**Methods:**

From January 2001 to December 2017, 3395 patients who underwent off-pump CABG (OPCAB) with BITA grafts were enrolled. The study population was stratified according to preoperative DM. The primary endpoint was cardiac death and the secondary endpoints were myocardial infarction (MI), revascularization, graft failure, stroke, postoperative wound infection, and a composite endpoint of cardiac death, MI, and revascularization. Multiple sensitivity analyses, including Cox proportional hazard regression and propensity-score matching analyses, were performed to adjust baseline differences.

**Results:**

After CABG, the DM group showed similar rates of cardiac death, MI, or revascularization and lower rates of graft failure at 10 years (DM vs. non-DM, 19.0% vs. 24.3%, hazard ratio [HR] 0.711, 95% confidence interval [CI] 0.549–0.925; *P* = 0.009) compared to the non-DM group. These findings were consistent after multiple sensitivity analyses. In the subgroup analysis, the well-controlled DM group, which is defined as preoperative hemoglobin A1c (HbA1c) of < 7%, showed lower postoperative wound infection rates (well-controlled DM vs. poorly controlled DM, 3.7% vs. 7.3%, HR 0.411, 95% CI 0.225–0.751; *P* = 0.004) compared to the poorly controlled DM group, which was consistent after propensity-score matched analysis.

**Conclusions:**

OPCAB with BITA grafts showed excellent and comparable long-term clinical outcomes in patients with and without DM. DM might have a protective effect on competition and graft failure of ITA. Strict preoperative hyperglycemia control with target HbA1c of < 7% might reduce postoperative wound infection and facilitate the use of BITA in CABG.

**Supplementary Information:**

The online version contains supplementary material available at 10.1186/s12933-022-01687-2.

## Background

Coronary artery bypass graft (CABG) is the recommended treatment for revascularization in patients with diabetes mellitus (DM) and coronary artery disease (CAD), especially in three-vessel diseases [1]. However, DM could worsen the prognosis after CABG because it frequently involves diffuse CAD that involves the left main, multi-vessel, or smaller vessels [2] and has higher chances of recurrent myocardial infarction (MI) and other comorbidities postoperatively [3, 4]. Additionally, the effect of DM on the long-term outcomes of CABG remained debatable based on published studies so far [5–9].

Importantly, various strategies exist for CABG, and when these are mixed in the same study, clarifying the effects of DM on CABG outcomes becomes difficult [5–7, 9]. Particularly, regarding graft selection, a wide variety of graft selection strategies exist in previous studies although internal thoracic artery (ITA) is proven patent in the long-term and is the graft choice for left anterior descending artery anastomosis [5–9]. Additionally, off-pump CABG (OPCAB) might remove the detrimental effects of cardiopulmonary bypass; however, a great deal of variability related to use of cardiopulmonary bypass exists for each center [5, 7]. Therefore, this study aimed to determine the effects of DM on patients who underwent OPCAB using bilateral ITA (BITA) grafts only.

Postoperative sternal wound infection is one of the most worrisome complications after CABG using BITA. Preoperative hemoglobin A1c (HbA1c) has been studied as a prognostic factor for postoperative wound infection in patients with DM who underwent cardiac surgery [10, 11]. The effect of HbA1c on postoperative morbidities, including mediastinal wound infection, was tried to define in this homogenous cohort.

## Methods

### Study design and populations

**Fig. 1:**
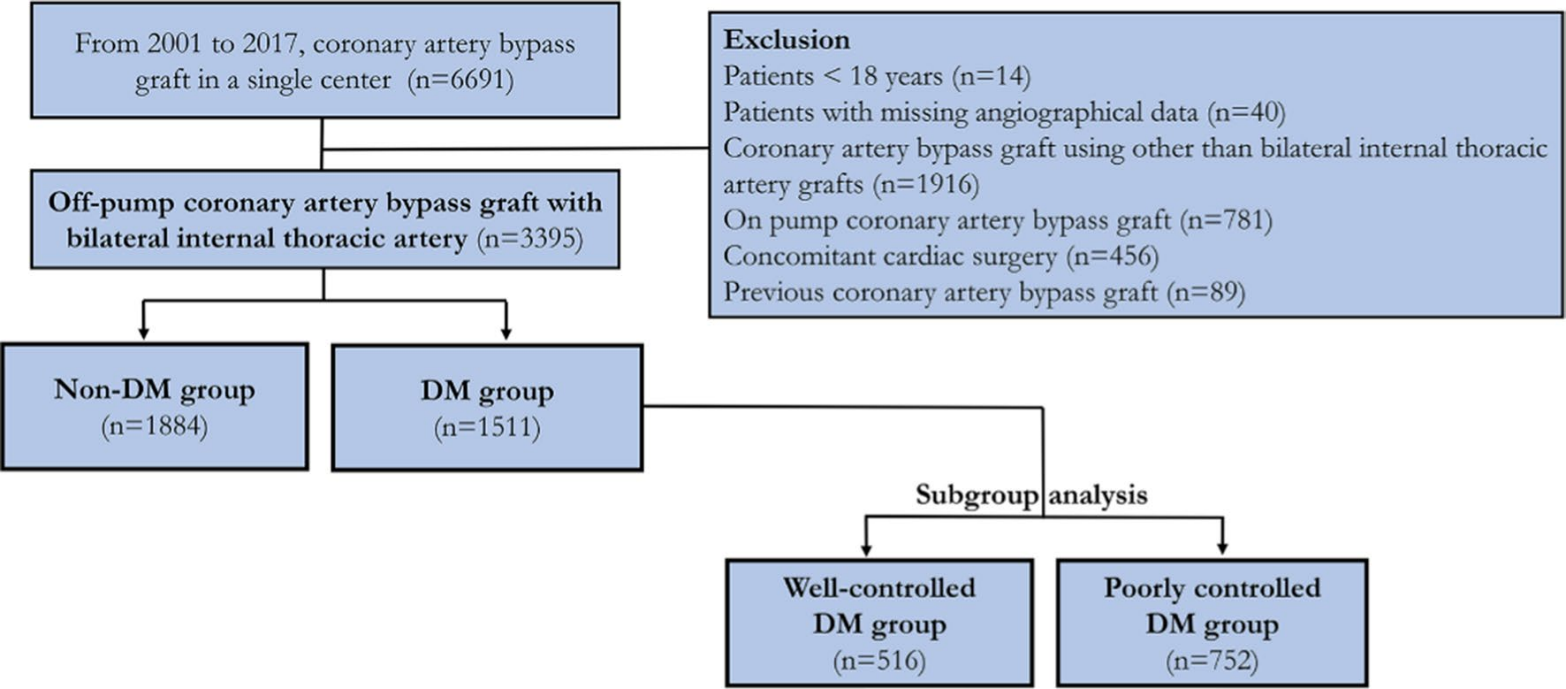
Study flow diagram. A total of 3395 patients who underwent OPCAB with a bilateral internal thoracic artery in a single tertiary center were enrolled. Patients were divided into non-DM and DM groups. Patients in the DM group were divided into well-controlled (HbA1c < 7%) and poorly controlled DM groups (HbA1c ≥ 7%). OPCAB indicates off-pump coronary artery bypass graft; DM: diabetes mellitus; HbA1c: hemoglobin A1c

This retrospective and the observational study included 6691 consecutive patients who underwent CABG using BITA in a single large tertiary center from January 2001 to December 2017. Patients younger than 18 years (n = 14), with missing angiographical data (n = 40), who underwent CABG other than BITA grafts (n = 1916), on-pump CABG (n = 781), concomitant heart surgery (n = 456), and had previous CABG history (n = 89) were excluded (Fig. 1). Patients were divided into two groups, the non-DM and DM groups. Patients in the DM group were divided into two subgroups, namely, the well-controlled (preoperative HbA1c of < 7%) and poorly controlled DM groups (HbA1c of ≥ 7%) [12].

The Institutional Review Board of Samsung Medical Center approved this study (SMC 2021-11-157, date of approval: December 1, 2021), which waived informed consent from individual patients because this retrospective study poses minimal risk for patients.

### Data collection and clinical follow-up

The baseline demographic, echocardiographic, laboratory, and follow-up clinical outcomes were retrospectively collected through medical record review. Extracted data were revalidated by our research coordinators and physicians for clarity.

The primary endpoint was cardiac death at 10 years postoperative and the secondary endpoints included MI, revascularization, graft failure, stroke, postoperative wound infection, and major adverse cardiovascular events (MACE; a composite endpoint of cardiac death, MI, and revascularization). The mean clinical outcome follow-up duration was 4.01 years. The mortality data for patients who were lost to follow-up were confirmed using the National Death Records. MI was defined as elevated cardiac troponin or myocardial band fraction of creatine kinase that is greater than the upper reference limit with concomitant ischemic symptoms or electrocardiography findings indicative of ischemia. Left ventricular dysfunction was classified as follow: normal (left ventricular ejection fraction more than 50%); mild dysfunction (left ventricular ejection fraction 40% to 49%); ≥ moderate dysfunction (left ventricular ejection fraction less than 40%). Graft patency was assessed using coronary computed tomography angiography or invasive coronary angiography, according to operator discretion (Additional file 1: Table S1). Grafts were evaluated following the FitzGibbon A, B, and O classification and interpreted by an independent cardiology expert who was blinded to treatment strategy. Postoperative early clinical outcomes included clinical outcomes within 30 days of the surgery. Outcome variables were defined according to the definition of the Society of Thoracic Surgeons Adult Cardiac Surgery Database version 4.20 [13].

### Surgical procedure and perioperative management

The surgical technique for OPCAB was performed following relevant standard guidelines [1, 14]. and described in our earlier report in detail [15]. BITA was prepared using the skeletonization technique. After opening the pericardium, the right ITA was anastomosed to the left side of the left ITA as a Y-composite graft. Achieving complete revascularization of all vessels with a 1-mm or larger diameter and 50% or more angiographic diameter stenosis was intended in all patients. The left ITA was first anastomosed to the left anterior descending artery and its branches, and the right ITA was sequentially anastomosed to branches of the circumflex artery. The right coronary territory was revascularized last. The quality of the anastomosis was assessed by transit-time flow measurement with a Transsonic Flowmeter (Transonic Systems, Ithaca, NY).

Strict glycemic control before, during, and after the surgery has been implemented under standard institutional protocols. Target blood glucose control in patients with DM was set to < 180 mg/dL [12, 16] by intermittent regular insulin injection (Humulin; Eli Lilly and Company, Indianapolis, Ind) intraoperatively and continuous regular insulin infusion postoperatively in the intensive care unit (ICU). Blood glucose level was measured every 1 h in the operative room and every 4 h in the ICU. If the ideal glycemic control was not made, glucose concentration was checked at an interval of 2 h until the target level was achieved in the ICU.

Guideline-directed medical therapy including a combination of antiplatelet agents, beta-blockers, statins, and angiotensin-converting enzyme (ACE) inhibitors and/or angiotensin receptor blockers (ARBs) was applied to all patients undergoing CABG, unless contraindicated [1, 17].

### Statistical analysis

Descriptive statistics for categorical variables were reported as frequency and percentage, whereas continuous variables were reported as mean ± standard deviation or median (range). Categorical variables were compared between the groups using the χ^2^ test or Fisher’s exact test, whereas continuous variables were compared using the two-sample t-tests or the Wilcoxon rank-sum test. Continuous data were checked for distribution normality using the Shapiro–Wilk test and graphical methods. The cumulative incidence of clinical events is estimated using a Fine–Gray model to account for the competing risk of death from non-cardiovascular causes.

Multiple sensitivity analyses, including multivariable Cox proportional hazard regression and propensity-score matched analyses, were performed to reduce biased effects. The Cox proportional hazard regression model considered variables that were significant in the univariable analysis or clinically relevant in the multivariable analysis. Variables included age, sex, body mass index, DM, HbA1c, an initial diagnosis of ischemic heart disease, hypertension, dyslipidemia, smoking history, end-stage renal disease requiring dialysis, previous cerebrovascular accident, abdominal aortic aneurysm, peripheral arterial disease, chronic obstructive pulmonary disease, peak troponin I, peak creatine kinase-MB, glomerular filtration rate, ejection fraction, left ventricular dysfunction, 3 vessel disease, left main involvement, and urgent surgery. The backward elimination method was used for model selection in the multivariable analysis. The results were reported as hazard ratio (HR) and 95% confidence interval (CI).

Propensity-score analyses were used to adjust covariate differences between the two groups. The variables included in the propensity-score model were as follows: age, sex, body mass index, angina, hypertension, dyslipidemia, previous stroke history, chronic kidney disease, chronic obstructive pulmonary disease, smoking history, left ventricular dysfunction, preoperative significant mitral valve regurgitation, urgent surgery, the number of anastomoses, and the number of diseased coronary arteries. A total of 1393 patients in the non-DM group and 1393 patients in the DM group were matched in a 1:1 manner using nearest-neighbor matching. Additionally, for subgroup analysis, a total of 452 patients in the well-controlled DM group and 452 patients in the poorly controlled DM group were matched in a 1:1 manner using nearest-neighbor matching. The balance between the two groups after propensity-score matching was assessed by calculating the standardized mean difference (SMD) between selected variables, with an SMD of < 0.20 suggesting an appropriate balance (Additional file 2: Fig. S1, Additional file 1: Tables S2 and S3).

All statistical tests were two-sided, with an alpha level of 0.05. Statistical analysis was performed using Statistical Package for the Social Sciences software (version 25.0, SPSS, Chicago, IL, USA) and R statistical software (version 4.0.2; R Foundation of Statistical Computing, Vienna, Austria).

## Results

### Baseline characteristics

A total of 3395 patients underwent CABG during the study period, including 1511 (44.5%) patients with DM and 1884 (55.4%) patients without DM (Fig. 1). The mean age of the patients was 63.3 years and 2619 (77.2%) patients were males. Additionally, 1898 (55.9%) patients had acute coronary syndrome, and 683 (20.1%) had left ventricular dysfunction. The mean number of anastomosis during CABG was 3.97 and postoperative guideline-directed medications, including aspirin, P2Y12 inhibitors, beta-blockers, ACE inhibitors or ARB, and statins were prescribed in 3300 (97.2%), 2046 (60.2%), 2530 (74.5%), 999 (29.4%), and 2721 (80.1%) patients, respectively.

Patients with DM were older (64.16 years vs. 62.62 years, *P* < 0.001) and more likely to have hypertension, smoking history, an end-stage renal disease requiring dialysis, previous cerebrovascular accident, abdominal aortic aneurysm, and peripheral arterial disease compared to patients without DM (Table 1). Additionally, patients with DM had a higher proportion of left ventricular dysfunction (25% vs. 16%, *P* < 0.001) and three-vessel diseases (75.6% vs. 64.8%, *P* < 0.001) and a bigger number of anastomosis (4.10 vs. 3.87, *P* < 0.001) during CABG than those without DM.

**Table 1:** Baseline and operative characteristics between the non-DM and DM groups

	Non-DM group (n = 1884)	DM group (n = 1511)	*P*-value
Demographics			
Age, years	62.62 ± 10.29	64.16 ± 8.80	< 0.001
Male sex	1505 (79.8%)	1114 (73.7%)	< 0.001
Body mass index**,** kg/m^2^	24.82 ± 2.90	24.65 ± 3.09	0.120
Cardiovascular risk factors			
Hypertension	1081 (57.4%)	1090 (72.1%)	< 0.001
Dyslipidemia	546 (28.9%)	464 (30.7%)	0.291
Smoking history	649 (34.5%)	455 (30.1%)	0.008
End-stage renal disease requiring dialysis	30 (1.59%)	92 (6.1%)	< 0.001
Previous cerebrovascular accident	198 (10.5%)	246 (16.2%)	< 0.001
Abdominal aortic aneurysm	28 (1.5%)	4 (0.2%)	< 0.001
Peripheral arterial disease	104 (5.5%)	127 (8.4%)	0.001
Chronic obstructive pulmonary disease	46 (2.4%)	27 (1.8%)	0.235
NYHA functional class of ≥ 3	126 (6.7%)	149 (9.8%)	0.001
CCS functional class of ≥ 3	317 (16.8%)	265 (17.5%)	0.616
Initial clinical presentation			
Stable ischemic heart disease	829 (44.0%)	668 (44.2%)	0.300
Unstable angina	794 (42.1%)	608 (40.2%)	
Acute myocardial infarction	261 (13.8%)	235 (15.6%)	
Laboratory data			
Peak troponin I, ng/mL	0.027 (0.006–0.15)	0.032 (0.007–0.18)	0.125
Peak CK-MB, ng/mL	1.19 (0.65–2.13)	1.31 (0.76–2.39)	0.002
Hemoglobin, g/dL	13.49 ± 1.67	12.66 ± 1.85	< 0.001
White blood cell count,/mm^3^	6.85 ± 1.87	6.93 ± 1.91	0.235
Creatinine, mg/dL	0.91 (0.8–1.07)	0.99 (0.82–1.22)	< 0.001
GFR< 60 mL/min/1.73m^2^	472 (25.1%)	619 (41.0%)	< 0.001
Total cholesterol, mg/dL	167.09 ± 43.13	155.73 ± 41.13	< 0.001
Low-density lipoprotein, mg/dL	109.29 ± 40.62	97.29 ± 36.60	< 0.001
High-density lipoprotein, mg/dL	43.49 ± 11.06	41.09 ± 10.60	< 0.001
C-reactive protein, mg/dL	0.17 (0.06–0.52)	0.15 (0.06–0.51)	0.493
Preoperative echography			
Ejection fraction, %	58.96 ± 10.78	56.46 ± 12.32	< 0.001
Left ventricular dysfunction			< 0.001
Normal	1580 (83.8%)	1133 (74.9%)	
Mild dysfunction	188 (9.9%)	198 (13.1%)	
≥ Moderate dysfunction	116 (6.1%)	180 (11.9%)	
MR more than mild	41 (2.2%)	39 (2.6%)	0.510
AR more than mild	7 (0.4%)	5 (0.3%)	> 0.999
TR more than mild	16 (0.9%)	9 (0.6%)	0.511
Preoperative coronary angiography			
Left main disease	504 (26.8%)	318 (21.1%)	< 0.001
3-vessel disease	1221 (64.8%)	1142 (75.6%)	< 0.001
2-vessel disease	634 (33.7%)	364 (24.1%)	< 0.001
1-vessel disease	29 (1.5%)	5 (0.3%)	0.001
Operative characteristics			
Type of surgery			0.169
Elective	1839 (97.6%)	1486 (98.4%)	
Urgent	45 (2.4%)	25 (1.75%)	
Number of anastomoses	3.87 ± 1.07	4.10 ± 1.02	< 0.001
Graft			
Left internal thoracic artery			
In situ graft	1863 (98.9%)	1471 (97.4%)	0.001
Composite graft	65 (3.5%)	63 (4.2%)	0.316
Free graft	10 (0.5%)	23 (1.5%)	0.006
Right internal thoracic artery			
In situ graft	32 (1.7%)	29 (1.92%)	0.725
Composite graft			0.264
1	1756 (93.2%)	1387 (91.8%)	
2	53 (2.8%)	55 (3.6%)	
Free graft	63 (3.3%)	60 (4.0%)	0.379
Medications			
Aspirin	1879 (99.7%)	1500 (99.3%)	0.075
P2Y12 inhibitors*	111 (59.0%)	935 (61.9%)	0.104
Beta-blockers	1381 (73.3%)	1149 (76.0%)	0.075
ACE inhibitors or ARB	516 (27.4%)	483 (32.0%)	0.004
Statins	1507 (80.0%)	1214 (80.3%)	0.829

Data are expressed as number (%), median (interquartile range), or mean ± SD values

*P2Y12 inhibitors included clopidogrel, ticagrelor, and prasugrel

*DM* diabetes mellitus, *NYHA* New York Heart Association, *CCS* Canadian Cardiovascular Society Angina Score, *CK-MB* creatine kinase-MB, *GFR* glomerular filtration rate, *MR* mitral regurgitation, *AR* aortic regurgitation, *TR* tricuspid regurgitation, *ACE* angiotensin-converting enzyme, *ARB* angiotensin receptor blocker

### Clinical outcomes

Incidences of postoperative mortality, bleeding requiring reoperation, and graft-related reoperation were 0.32%, 1.08%, and 0.38%, respectively. The DM group had higher rates of acute kidney injury (8.57% vs. 3.83%, *P* < 0.001), wound infection (5.69% vs. 1.70%, *P* < 0.001), and repeated wound infection (1.0% vs. 0.4%, *P* = 0.043) than in the non-DM group in the early postoperative period (Table 2).

**Table 2:** Postoperative outcomes according to preoperative DM

	Non-DM group	DM group	Unadjusted			Multivariable adjusted*			Propensity-score matched		
			HR	95% CI	*P*-value	HR	95% CI	*P*-value	HR	95% CI	*P-*value
Early clinical outcomes (< 30 days)	N = 1884	N = 1511							N = 2786 (1393 Pairs)		
Early mortality	7 (0.5%)	4 (0.3%)	0.716	0.209–2.450	0.764				0.570	0.167–1.952	0.504
Bleeding requiring reoperation	19 (1.0%)	18 (1.2%)	1.183	0.619–2.263	0.731				2.266	0.982–5.230	0.076
Graft related reoperation	7 (0.4%)	6 (0.4%)	1.069	0.359–3.188	> 0.999				1.000	0.322–3.108	> 0.999
Myocardial infarction	2 (0.1%)	0 (0.0%)	NA	NA	NA				NA	NA	NA
Stroke	10 (0.5%)	12 (0.8%)	1.500	0.646–3.482	0.462				1.336	0.561.3.181	0.661
Atrial fibrillation	51 (2.7%)	50 (3.3%)	1.230	0.828–1.828	0.355				1.025	0.662–1.587	> 0.999
Acute kidney injury	63 (3.8%)	116 (8.6%)	2.354	1.717–3.228	< 0.001				1.877	1.322–2.666	0.001
Dialysis	3 (0.2%)	16 (1.6%)	6.811	1.979–23.443	0.001				3.744	1.041–13.466	0.057
Wound infection	32 (1.7%)	86 (5.7%)	3.493	2.314–5.272	< 0.001				3.202	2.026–5.059	< 0.001
Repeated wound infection	7 (0.4%)	15 (1.0%)	2.689	1.093–6.611	0.043				2.178	0.825–5.746	0.167
Clinical outcomes at 10 year											
Cardiac death	30 (3.3%)	30 (5.1%)	1.407	0.848–2.358	0.186	0.885	0.451–1.734	0.722	0.954	0.544–1.669	0.865
Myocardial infarction	27 (4.6%)	19 (5.0%)	1.060	0.581–1.931	0.846	0.815	0.845–1.077	0.594	0.998	0.528–1.921	0.996
Repeat revascularization	65 (9.6%)	47 (12.8%)	1.079	0.733–1.587	0.692	1.241	0.838–1.838	0.281	1.137	0.742–1.741	0.545
MACE	77 (17.1%)	99 (13.6%)	1.141	0.846–1.546	0.387	1.081	0.794–1.472	0.620	1.042	0.751–1.455	0.804
Stroke	42 (5.9%)	46 (7.8%)	1.580	1.032–2.420	0.031	1.360	0.875–2.112	0.170	1.305	0.832–2.047	0.236
Graft related outcomes at 10 year											
ITA graft failure^†^	172 (24.3%)	87 (19.0%)	0.711	0.549–0.925	0.009	0.767	0.592–0.994	0.045	0.708	0.536–0.935	0.013
FitzGibbon grade B	22 (3.0%)	13 (2.9%)	0.813	0.407–1.642	0.556	0.899	0.451–1.791	0.762	0.922	0.437–1.980	0.832
FitzGibbon grade O	149 (21.7%)	74 (16.6%)	0.695	0.526–0.918	0.010	0.752	0.568–0.996	0.046	0.685	0.511–0.919	0.011
LITA graft failure	66 (8.6%)	38 (7.6%)	0.794	0.532–1.193	0.257	0.865	0.579–1.294	0.481	0.773	0.506–1.191	0.235
RITA graft failure	115 (18.1%)	57 (13.4%)	0.696	0.507–0.957	0.025	0.726	0.528–0.999	0.049	0.703	0.504–0.981	0.038

Data are expressed as numbers (%). Cumulative incidence of events was presented as Kaplan–Meier estimates

*DM* diabetes mellitus, *HR* hazard ratio, *CI* confidence interval, *NA* not applicable, *MACE* major adverse cardiovascular event, *ITA* internal thoracic artery, *LITA* left internal thoracic artery, *RITA* right internal thoracic artery

*Multivariable analysis was not performed for early clinical outcomes due to a small number of events

^†^FitzGibbon grade A indicated that the graft was patent with ≤ 50% stenosis. FitzGibbon grade B indicated that the extent of graft stenosis was > 50% but not occluded. FitzGibbon grade O indicated total graft occlusion without contrast filling. Herein, graft failure was defined as FitzGibbon grade B or O

In the long-term, no differences were found in the rates of cardiac death, MI, repeat revascularization, stroke, and MACE between the DM and non-DM groups (Table 2).

**Fig. 2:**
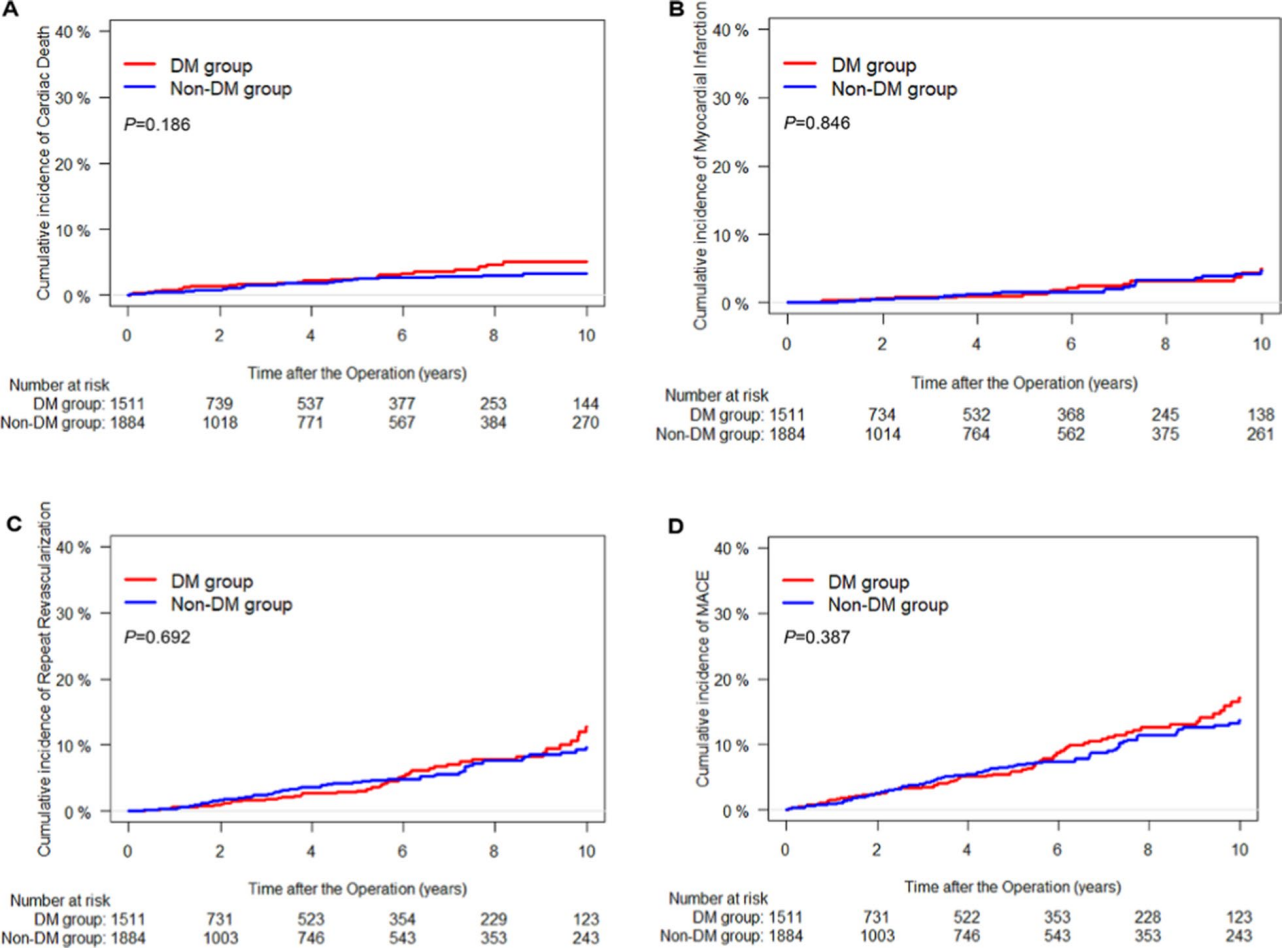
Cumulative incidence curves for cardiac death (**A**), myocardial infarction (**B**), repeat revascularization (**C**), and MACE (**D**) according to DM. Non-cardiac death was accounted as a competing event in the Fine–Gray model. MACE indicates major adverse cardiovascular and cerebrovascular events; DM: diabetes mellitus

**Fig. 3:**
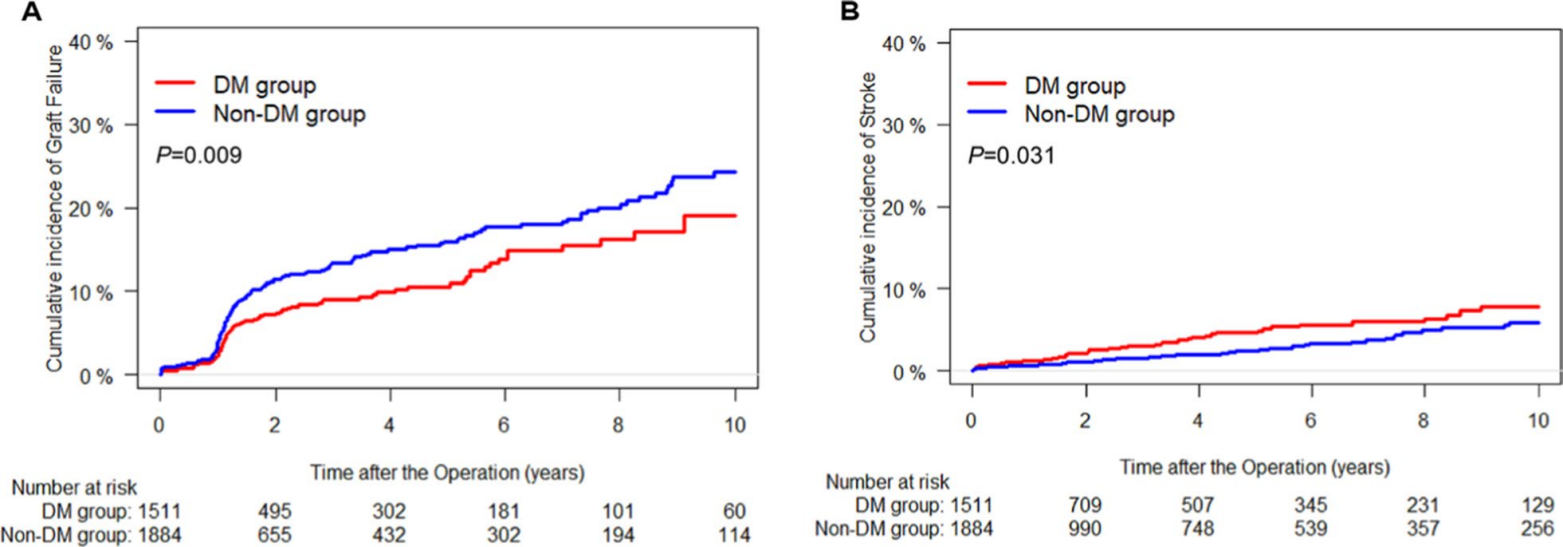
Cumulative incidence curves for graft failure (**A**) and stroke (**B**) according to DM. Non-cardiac death was accounted as a competing event in the Fine–Gray model. DM indicates diabetes mellitus

Multivariable Cox proportional hazard models and propensity-score matched analyses consistently showed similar results for patients in both groups (Table 2, Figs. 2 and 3).

### ITA graft outcomes

The mean time from surgery to graft failure was 2.60 ± 2.99 years. Patients in the DM group had lower rates of ITA graft failure (19.0% vs. 24.3%, HR = 0.711, *P* = 0.009), ITA graft failure with FitzGibbon grade O (16.6% vs. 21.7%, HR = 0.695, *P* = 0.010), and right ITA graft failure (13.4% vs. 18.1%, HR = 0.696, *P* = 0.025). Multivariable Cox proportional hazard models and propensity-score matched analyses consistently showed similar results for patients in both groups (Table 2, Fig. 3A).

### Subgroup analysis

Compared with patients with poorly controlled DM, patients with well-controlled DM were older and more likely to have hypertension, end-stage renal disease that requires dialysis, and previous cerebrovascular accidents (Table 3). However, no statistical differences were seen between the two groups regarding preoperative echocardiography, coronary angiography, and operative characteristics. In the long-term, there were no differences found in the rates of cardiac death, MI, repeat revascularization, stroke, and MACE between the well-controlled DM and poorly controlled DM groups (Additional file 2: Figs. S2 and S3). However, patients with well-controlled DM had a lower proportion of postoperative wound infection (3.7% vs. 7.3%, HR = 2.064, 95% CI 1.210–2.521, *P* = 0.0007; Table 4). Multivariable Cox proportional hazard models and propensity-score matched analyses consistently showed similar results for patients in the well-controlled and poorly controlled DM groups.

**Table 3:** Baseline and operative characteristics between well-controlled vs. poorly controlled DM groups

Variables	Well-controlled DM group (n = 516)	Poorly-controlled DM group (n = 752)	*P* value
Demographics			
Age, years	66.07 ± 8.91	63.47 ± 8.61	< 0.001
Male sex	397 (76.94%)	549 (73.01%)	0.130
Body mass index**,** kg/m^2^	24.56 ± 3.10	24.74 ± 3.07	0.322
Comorbidities			
Hypertension	411 (79.7%)	521 (69.28%)	< 0.001
Dyslipidemia	156 (30.2%)	256 (34.04%)	0.173
Smoking history	142 (27.5%)	240 (31.91%)	0.107
End-stage renal disease requiring dialysis	42 (8.1%)	41 (5.45%)	0.074
Previous cerebrovascular accident	99 (19.2%)	109 (14.49%)	0.032
Abdominal aortic aneurysm	2 (0.4%)	1 (0.13%)	0.743
Peripheral arterial disease	47 (9.1%)	62 (8.24%)	0.662
Chronic obstructive pulmonary disease	12 (2.3%)	12 (1.60%)	0.467
NYHA functional class of ≥ 3	39 (7.6%)	72 (9.57%)	0.251
CCS functional class of ≥ 3	58 (11.2%)	109 (14.49%)	0.110
Initial clinical presentation			0.699
Stable ischemic heart disease	223 (43.2%)	328 (43.6%)	
Unstable angina	219 (42.4%)	305 (40.6%)	
Acute myocardial infarction	74 (14.3%)	119 (15.82%)	
Laboratory data			
Peak troponin I, ng/mL	0.024 (0.006–0.18)	0.023 (0.006–0.18)	0.937
Peak CK-MB, ng/mL	1.35 (0.79–2.7)	1.28 (0.75–2.27)	0.134
Hemoglobin, g/dL	12.40 ± 1.91	12.79 ± 1.86	< 0.001
White blood cell count,/mm^3^	6.74 ± 1.87	7.08 ± 1.91	0.002
Creatinine, mg/dL	1 (0.82–1.26)	0.98 (0.81–1.2)	0.053
GFR of < 60 mL/min/1.73m^2^	234 (45.4%)	297 (39.49%)	0.044
Total cholesterol, mg/dL	149.80 ± 39.56	156.79 ± 42.59	0.004
Low-density lipoprotein, mg/dL	93.29 ± 36.34	97.19 ± 36.97	0.083
High-density lipoprotein, mg/dL	41.64 ± 10.66	41.07 ± 10.58	0.385
C-reactive protein, mg/dL	0.14 (0.05–0.46)	0.15 (0.06–0.48)	0.485
Preoperative echography			
Ejection fraction, %	57.51 ± 12.06	56.28 ± 12.40	0.079
Left ventricular dysfunction			0.115
Normal	407 (78.9%)	555 (73.80%)	
Mild dysfunction	56 (10.9%)	103 (13.70%)	
≥ Moderate dysfunction	53 (10.3%)	94 (12.50%)	0.286
MR more than mild	16 (3.1%)	15 (1.99%)	
AR more than mild	2 (0.4%)	2 (0.27%)	> 0.999
TR more than mild	1 (0.2%)	5 (0.66%)	0.433
Preoperative coronary angiography			
Left main disease	113 (21.9%)	152 (20.21%)	0.512
3-vessel disease	406 (78.7%)	579 (76.99%)	0.522
2-vessel disease	108 (20.9%)	170 (22.61%)	0.522
1-vessel disease	2 (0.4%)	3 (0.40%)	> 0.999
Operative characteristics			
Type of surgery			0.913
Elective	509 (98.6%)	740 (98.40%)	
Urgent	7 (1.4%)	12 (1.60%)	
Number of anastomoses	4.20 ± 0.98	4.18 ± 1.01	0.673
Graft			
Left internal thoracic artery			
In situ graft	498 (96.5%)	736 (97.87%)	0.195
Composite graft	21 (4.1%)	33 (4.39%)	0.893
Free graft	9 (1.7%)	11 (1.46%)	0.868
Right internal thoracic artery			
In situ graft	10 (1.9%)	10 (1.33%)	0.532
Composite graft			0.403
1	472 (91.5%)	697 (92.69%)	
2	20 (3.9%)	31 (4.12%)	
Free graft	18 (3.5%)	22 (2.93%)	0.689
Medications			
Aspirin	513 (99.4%)	746 (99.2%)	0.746
P2Y12 inhibitors*	379 (73.4%)	511 (68.0%)	0.039
Beta-blockers	413 (80.0%)	608 (80.9%)	0.719
ACE inhibitors or ARB	147 (28.5%)	232 (30.9%)	0.382
Statins	416 (80.6%)	587 (78.1%)	0.292

Data are expressed as numbers (%), median (interquartile range), or mean ± SD values

*P2Y12 inhibitors included clopidogrel, ticagrelor, and prasugrel

*DM* diabetes mellitus, *NYHA* New York Heart Association, *CCS* Canadian Cardiovascular Society Angina Score, *CK-MB* creatine kinase-MB, *GFR* glomerular filtration rate, *MR* mitral regurgitation, *AR* aortic regurgitation, *TR* tricuspid regurgitation, *ACE* angiotensin-converting enzyme, *ARB* angiotensin receptor blocker

**Table 4:** Postoperative outcomes according to preoperative HbA1c in DM patients

	Well-controlled DM group	Poorly controlled DM group	Unadjusted			Multivariable adjusted*			Propensity-score matched		
			HR	95% CI	*P*-value	HR	95% CI	*P*-value	HR	95% CI	*P-*value
Early outcomes (< 30 days)	N = 516	N = 752							N = 904 (452 Pairs)		
Early mortality	1 (0.3%)	1 (0.2%)	0.638	0.040–10.232	> 0.999				0.248	0.028–2.231	0.374
Bleeding requiring reoperation	7 (1.4%)	9 (1.2%)	0.881	0.326–2.380	> 0.999				1.169	0.390–3.507	> 0.999
Graft related reoperation	2 (0.4%)	1 (0.1%)	0.342	0.031–3.784	0.570				0.499	0.045–5.521	> 0.999
Myocardial infarction	0 (0%)	0 (0%)	NA	NA	NA				NA	NA	NA
Stroke	3 (0.6%)	8 (1.1%)	1.839	0.485–6.964	0.540				2.354	0.605–9.162	0.341
Atrial fibrillation	16 (3.1%)	30 (4.0%)	1.298	0.700–2.407	0.448				2.149	1.035–4.463	0.053
Acute kidney injury	40 (8.4%)	52 (7.5%)	0.892	0.581–1.372	0.659				1.114	0.666–1.864	0.696
Dialysis	8 (2.0%)	7 (1.3%)	0.641	0.230–1.782	0.436				0.926	0.308–2.784	> 0.999
Wound infection	19 (3.7%)	55 (7.3%)	2.064	1.210–3.521	0.007				2.430	1.331–4.434	0.004
Repeated wound infection	4 (0.8%)	9 (1.2%)	1.550	0.475–5.062	0.577				3.539	0.731–17.130	0.178
Long-term outcomes at 10 years											
Cardiac death	8 (5.4%)	10 (3.5%)	0.783	0.311–2.013	0.605	0.839	0.308–2.284	0.731	0.946	0.273–3.362	0.930
Myocardial infarction	6 (7.0%)	8 (5.7%)	0.783	0.273–2.302	0.650	0.938	0.320–0.750	0.906	1.274	0.404–4.114	0.679
Repeat revascularization	16 (16.9%)	15 (10.2%)	0.532	0.264–1.087	0.077	0.464	0.226–0.952	0.036	0.643	0.287–1.469	0.285
MACE	23 (20.3%)	26 (13.5%)	0.664	0.379–1.176	0.152	0.628	0.344–1.146	0.130	0.773	0.398–1.523	0.449
Stroke	14 (8.4%)	27 (10.6%)	1.245	0.652–2.409	0.506	1.416	0.730–2.747	0.303	1.668	0.815–3.468	0.161
Graft related outcomes at 10 years											
ITA graft failure^†^	20 (8.9%)	49 (20.1%)	1.457	0.868–2.470	0.154	1.327	0.787–2.236	0.288	1.093	0.616–1.965	0.755
FitzGibbon grade B	1 (0.3%)	8 (3.2%)	4.760	0.605–38.872	0.138	4.760	0.594–38.130	0.142	3.135	0.376–27.187	0.291
FitzGibbon grade O	19 (8.7%)	41 (17.5%)	1.283	0.747–2.228	0.366	1.173	0.679–2.024	0.567	0.983	0.536–1.824	0.956
LITA graft failure	10 (3.6%)	18 (7.3%)	1.101	0.513–2.402	0.805	0.985	0.453–2.142	0.970	0.702	0.282–1.782	0.447
RITA graft failure	13 (6.7%)	35 (14.9%)	1.582	0.829–3.019	0.156	1.333	0.702–2.533	0.380	1.269	0.637–2.567	0.498

Data are expressed as numbers (%). Cumulative incidence of events was presented as Kaplan–Meier estimates

*HbA1c* hemoglobin A1c, *DM* diabetes mellitus, *HR* hazard ratio, *CI* confidence interval, *NA* not applicable, *MACE* major adverse cardiovascular event, *ITA* internal thoracic artery, *LITA* left internal thoracic artery, *RITA* right internal thoracic artery

*Multivariable analysis was not performed for early clinical outcomes due to a small number of events

^†^FitzGibbon grade A indicated that the graft was patent with ≤ 50% stenosis. FitzGibbon grade B indicated that the extent of graft stenosis was > 50% but not occluded. FitzGibbon grade O indicated total graft occlusion without contrast filling. Herein, graft failure was defined as FitzGibbon grade B or O

### Multivariable analysis for outcomes

Preoperative dialysis (HR = 2.256, 95% CI 1.223–4.161, *P* = 0.009) and glomerular filtration rate of < 60 mL/min/1.73 m^2^ (HR = 1.684, 95% CI 1.188–2.387, *P* = 0.003) were significant predictors of MACE in the multivariable analysis (Table 5). Additionally, age at operation (HR = 0.986, 95% CI 0.974–0.998, *P* = 0.028) and DM (HR = 0.718, 95% CI 0.556–0.929, *P* = 0.011) were prognostic factors for postoperative graft failure. Female sex (HR = 0.359, 95% CI 0.241–0.510, *P* < 0.001), DM (HR = 3.264, 95% CI 2.158–4.938, *P* < 0.001), and HbA1c of < 7% (HR = 0.333, 95% CI 0.219–0.505, *P* = 0.001) were significant prognostic factors for postoperative wound infection.

**Table 5:** Independent predictors of outcomes

Variable	Multivariable-adjusted model	
	HR (95% CI)	*P-*value
MACE		
Dialysis	2.256 (1.223–4.161)	0.009
GFR of < 60 mL/min/1.73 m^2^	1.684 (1.188–2.387)	0.003
DM	1.067 (0.794–1.432)	0.665
HbA1c level	0.912 (0.793–1.050)	0.201
HbA1c level of < 7%	1.393 (0.914–2.121)	0.122
ITA graft failure		
Age	0.986 (0.974–0.998)	0.028
DM	0.718 (0.556–0.929)	0.011
HbA1c level	0.940 (0.846–1.046)	0.259
Postoperative wound infection		
Female sex	0.351 (0.241–0.510)	< 0.001
DM	3.264 (2.158–4.938)	< 0.001
HbA1c of < 7%	0.333 (0.219–0.505)	0.001

*HR* hazard ratio, *CI* confidence interval, *MACE* major adverse cardiovascular event, *GFR* glomerular filtration rate, *DM* diabetes mellitus, *HbA1c* hemoglobin A1c, *ITA* internal thoracic artery

## Discussion

This study revealed the following key findings: (1) DM had no significant impact on long-term clinical outcomes, including cardiac death and MACE after OPCAB with BITA, (2) patients with DM had lower rates of postoperative graft failure compared to those without DM, and (3) patients with well-controlled DM, which is defined as preoperative HbA1c of < 7%, had a lower proportion of postoperative wound infection compared to those with poorly controlled DM.

Patients with DM who undergo intervention for CAD might have a poorer prognosis than those without DM, although the impact of DM on long-term mortality remained controversial [5, 6, 8, 18]. Patients with DM have more left main or multi-vessel CAD with a diffuse disease that involves smaller vessels [2], and they have a greater atherosclerotic burden and increased number of lipid-rich plaques, which are prone to rupture [19–21].

However, long-term survival and MACE between the DM group and non-DM groups in this study were not statistically different due to several reasons. First, CABG might have a protective effect against recurrent MI in patients with DM [22]. In complex CAD, the most common cause of mortality might be MI-related death [23], which tends to cluster within the proximal third of major coronary vessels [24]. CABG might provide the coverage of anatomic zones at risk for MI [25] and have the effect of preventing recurrent MI in patients with DM because graft insertion sites seem to be consistently located distal to acute thrombosis sites in patients with CABG.

Another important question is the influence of DM on the long-term patency of ITA grafts. Raza et al. [26] investigated postoperative angiograms quantifying stenosis in ITA grafts in patients who underwent primary isolated CABG and found that DM did not influence the long-term patency of bypass grafts. Early ITA graft patency was even better in patients with DM than in those without DM in the same study [26]. Additionally, Ralf et al. found that not having DM was a predictor of ITA graft failure and which might have been caused by competitive flow in their well-designed study with completed angiographic follow-up [27]. ITA grafts can autoregulate depending on flow requirements; thus, they may close due to competitive blood flow in the native vessel, especially in patients without DM [28–30]. In the current study, the non-DM group had higher rates of graft failure than the DM group and most of the cases were within the early phase, which is consistent with previously described studies. We might infer that most graft failures might have been caused by competition, not by surgical occlusion or atherosclerosis from the observation that most graft failures occurred in the early phase, not in the immediate postoperative or late phase. Additionally, most graft failures occurred in the form of occlusion rather than stenosis, and in RITA rather than in LITA, which might have a lower chance of competition due to the low number of anastomosis per graft [27] compared to RITA. Severe stenotic coronary disease in DM might have a protective effect on graft patency, which might need further investigation. Fractional flow reserve-guided CABG, which is now in practice at our center, might avoid graft failure caused by competition and enhance long-term outcomes, especially in patients without DM [31, 32].

HbA1c, which reflects the mean glycemia over the previous 8–12 weeks [33, 34], has been investigated as a parameter exerting an adverse influence on outcomes in patients with DM who underwent cardiac surgery [10, 11]. In this study, postoperative wound infection, which is the major drawback of using bilateral ITA grafts for CABG in patients with DM, was significantly lower in the well-controlled DM group, although without differences regarding cardiac mortality and MACE between the well-controlled and poorly controlled DM groups. The preoperative strict control of hyperglycemia might lower the incidence of postoperative wound infection and facilitate the use of BITA when performing CABG.

The present study has the following limitations. First, this is a retrospective study in a single tertiary center and might have selection bias. Second, we did not routinely perform invasive coronary angiography or coronary computed tomography angiography after CABG and the graft-related outcomes might be overestimated. Last, the adherence of medical treatment was lower than expected and not considered in the present analysis. However, important strengths of our study include its large sample size, homogeneous group using identical strategy in performing CABG, and strict adjustment for confounding factors using multiple sensitivity analysis.

In conclusion, OPCAB with BITA grafts showed excellent and comparable long-term clinical outcomes in patients with and without DM. DM might have a protective effect on competition and graft failure of ITA, and strict preoperative control of hyperglycemia with target HbA1c of < 7% might reduce postoperative wound infection and facilitate the use of BITA in CABG.

## Supplementary Information

**Additional file 1: Table S1.** Graft evaluation modalities after CABG according to DM. **Table S2.** Baseline and operative characteristics in propensity-matched population between the Non-DM group and DM group. **Table S3.** Baseline and operative characteristics in propensity-matched population between the well-controlled vs. poorly controlled DM groups.

**Additional file 2: Figure S1.** Love plots for propensity score matching (A) between DM and non-DM groups and (B) between well-controlled and poorly controlled DM groups. **Figure S2.** Cumulative incidence curves for cardiac death (A), myocardial infarction (B), repeat revascularization (C), and MACE (D) according to preoperative HbA1c. Non-cardiac death was accounted as a competing event in the Fine-Gray model. **Figure S3.** Cumulative incidence curves for graft failure (A) and stroke (B) according to preoperative HbA1c. Non-cardiac death was accounted as a competing event in the Fine-Gray model.

## Data Availability

The datasets used and analyzed during the current study are available from the corresponding author on reasonable request.

## Abbreviations

BITA: Bilateral internal thoracic artery
CABG: Coronary artery bypass grafting
CAD: Coronary artery disease
HbA1c: Hemoglobin A1c
ITA: Internal thoracic artery
ICU: Intensive care unit
DM: Diabetes mellitus
MI: Myocardial infarction
MACE: Major adverse cardiovascular events
OPCAB: Off-pump coronary artery bypass grafting
