# Supplementary material for: Impact of diabetes mellitus on long-term clinical and graft outcomes after off-pump coronary artery bypass grafting with pure bilateral skeletonized internal thoracic artery grafts
Source: Cardiovasc Diabetol. 2022 Nov 15;21:243. doi: 10.1186/s12933-022-01687-2 (PMC9667562; doi:10.1186/s12933-022-01687-2)
Supplement: Supplementary file 1 — Additional file 1: Table S1. Graft evaluation modalities after CABG according to DM. Table S2. Baseline and operative characteristics in propensity-matched population between the Non-DM group and DM group. Table S3. Baseline and operative characteristics in propensity-matched population between the well-controlled vs. poorly controlled DM groups. [file 12933_2022_1687_MOESM1_ESM.docx]

**Table S1.** Graft evaluation modalities after CABG according to DM

|  | **Non-DM Group** | **DM Group** | ***P*-value** |
| --- | --- | --- | --- |
| **Evaluation modality**  CCTA  CAG  Not evaluated | 642 (34.1%)  393 (20.9%)  848 (45.0%) | 422 (27.9%)  287 (19.0%)  802 (53.1%) | <0.001 |

Data are expressed as numbers (%).

CABG indicates coronary artery bypass grafting, *DM* diabetes mellitus, *CCTA* coronary computed tomography angiography, *CAG* coronary angiography.

**Table S2.** Baseline and operative characteristics in propensity-matched population between the non-DM group and DM group

| **Variables** | **Non-DM Group (n = 1393)** | **DM Group**  **(n = 1393)** | **SMD** |
| --- | --- | --- | --- |
| **Demographics**  Age, years  Male sex  Body mass index**,** kg/m^2^ | 63.89 ± 9.80  1066 (76.5%)  24.79 ± 2.92 | 64.06 ± 8.89  1048 (75.2%)  24.69 ± 3.07 | 0.018  0.030  0.033 |
| **Cardiovascular risk factors**  Hypertension  Dyslipidemia  Smoking history  End-stage renal disease requiring dialysis  Previous cerebrovascular accident  Abdominal aortic aneurysm  Peripheral arterial disease  Chronic obstructive pulmonary disease  NYHA functional class of ≥3  CCS functional class of ≥3  **Initial clinical presentation**  Stable ischemic heart disease  Unstable angina  Acute myocardial infarction | 967 (69.4%)  436 (31.3%)  446 (32.0%)  30 (2.2%)  187 (13.4%)  23 (1.7%)  89 (6.4%)  25 (1.8%)  99 (7.1%)  223 (16.0%)  628 (45.1%)  572 (41.1%)  193 (13.9%) | 976 (70.1%)  431 (30.9%)  433 (31.1%)  41 (2.9%)  209 (15.0%)  4 (0.3%)  89 (6.4%)  26 (1.9%)  126 (9.1%)  241 (17.3%)  622 (44.7%)  572 (41.1%)  199 (14.3%) | 0.014  0.008  0.020  0.050  0.045  0.140  0.064  0.005  0.071  0.035  0.013 |
| **Laboratory data**  Peak troponin I, ng/mL  Peak CK-MB, ng/mL  Hemoglobin, g/dL  White blood cell count, /mm^3^  Creatinine, mg/dL  GFR < 60mL/min/1.73m^2^  Total cholesterol, mg/dL  Low-density lipoprotein, mg/dL  High-density lipoprotein, mg/dL  C-reactive protein, mg/dL  **Preoperative echography**  Ejection fraction, %  Left ventricular dysfunction  Normal  Mild dysfunction  ≥Moderate dysfunction  MR more than mild  AR more than mild  TR more than mild  **Preoperative coronary angiography**  Left main disease  3-vessel disease  2-vessel disease  1-vessel disease | 1.28 ± 5.52  4.52 ± 15.41  13.39 ± 1.70  6.86 ± 1.89  1.08 ± 1.09  396 (28.4%)  165.72 ± 42.61  107.74 ± 40.11  43.61 ± 11.44  0.71 ± 1.94  58.39 ± 11.38  1121 (80.5%)  163 (11.7%)  109 (7.8%)  28 (2.0%)  7 (0.5%)  13 (0.9%)  305 (21.9%)  1010 (72.5%)  380 (27.3%)  3 (0.2%) | 1.78 ± 9.31  4.20 ± 16.35  12.79 ± 1.81  6.91 ± 1.9  1.22 ± 1.24  524 (37.6%)  155.98 ± 40.51  97.85 ± 36.50  41.02 ± 10.40  0.70 ± 1.84  57.44 ± 11.61  1091 (78.3%)  175 (12.6%)  127 (9.1%)  31 (2.2%)  5 (0.4%)  7 (0.5%)  306 (22.0%)  1043 (74.9%)  345 (24.8%)  5 (0.4%) | 0.065  0.020  0.342  0.028  0.120  0.196  0.234  0.258  0.237  0.010  0.082  0.056  0.015  0.022  0.051  0.002  0.054  0.057  0.027 |
| **Operative characteristics**  Type of surgery  Elective  Urgent  Number of anastomoses  Graft  Left internal thoracic artery  In situ graft  Composite graft  Free graft  Right internal thoracic artery  In situ graft  Composite graft  1  2  Free graft  Medications  Aspirin  P2Y12 inhibitors^*^  Beta-blockers  ACE inhibitors or ARB  Statins | 1370 (98.4%)  23 (1.7%)  4.05 ± 1.03  1378 (98.9%)  56 (4.0%)  6 (0.4%)  21 (1.5%)  1297 (93.1%)  44 (3.2%)  46 (3.3%)  1390 (99.8%)  833 (59.8%)  1032 (74.1%)  404 (29.0%)  1096 (78.7%) | 1369 (98.3%)  24 (1.7%)  4.06 ± 1.02  1364 (97.9%)  54 (3.9%)  16 (1.2%)  28 (2.0%)  1280 (91.9%)  50 (3.6%)  51 (3.7%)  1384 (99.4%)  857 (61.5%)  1061 (76.2%)  434 (31.2%)  1132 (81.3%) | 0.006  0.016  0.081  0.007  0.081  0.038  0.047  0.020  0.066  0.051  0.060  0.047  0.074 |

Data are expressed as numbers (%) or mean ± SD values.

^*^P2Y12 inhibitors included clopidogrel, ticagrelor, and prasugrel.

*DM* diabetes mellitus, *SMD* standardized mean difference, *NYHA* New York Heart Association, *CCS* Canadian Cardiovascular Society Angina Score, *CK-MB* Creatine kinase-MB, *GFR* glomerular filtration rate, *MR* mitral regurgitation, *AR* aortic regurgitation, *TR* tricuspid regurgitation, *ACE* angiotensin-converting enzyme, *ARB* angiotensin receptor blocker

**Table S3.** Baseline and operative characteristics in propensity-matched population between the well-controlled vs. poorly controlled DM groups

|  | **Well-controlled**  **DM group (n = 452)** | **Poorly-controlled**  **DM group (n = 452)** | | **SMD** | |
| --- | --- | --- | --- | --- | --- |
| **Demographics**  Age, years  Male sex  Body mass index**,** kg/m^2^ | 65.02 ± 8.81  336 (74.34%)  24.65 ± 3.15 | 65.34 ± 7.85  339 (75.00%)  24.59 ± 2.98 | 0.037  0.015  0.021 | |  |
| **Comorbidities**  Hypertension  Dyslipidemia  Smoking history  End-stage renal disease requiring dialysis  Previous cerebrovascular accident  Abdominal aortic aneurysm  Peripheral arterial disease  Chronic obstructive pulmonary disease  NYHA functional class of ≥3  CCS functional class of ≥3  **Initial clinical presentation**  Stable ischemic heart disease  Unstable angina  Acute myocardial infarction | 349 (77.21%)  148 (32.74%)  130 (28.76%)  29 (6.42%)  75 (16.59%)  1 (0.22%)  38 (8.41%)  9 (1.99%)  35 (7.74%)  50 (11.06%)  199 (44.0%)  189 (41.8%)  64 (14.16%) | 342 (75.66%)  137 (30.31%)  134 (29.65%)  32 (7.08%)  81 (17.92%)  0 (0.0%)  45 (9.96%)  8 (1.77%)  44 (9.73%)  69 (15.27%)  198 (43.8%)  188 (41.6%)  66 (14.60%) | 0.036  0.052  0.019  0.026  0.035  0.067  0.054  0.026  0.071  0.125  0.013 | |  |
| **Laboratory data**  Peak troponin I, ng/mL  Peak CK-MB, ng/mL  Hemoglobin, g/dL  White blood cell count, /mm^3^  Creatinine, mg/dL  GFR of <60mL/min/1.73m^2^  Total cholesterol, mg/dL  Low-density lipoprotein, mg/dL  High-density lipoprotein, mg/dL  C-reactive protein, mg/dL  **Preoperative echography**  Ejection fraction, %  Left ventricular dysfunction  Normal  Mild dysfunction  ≥Moderate dysfunction  MR more than mild  AR more than mild  TR more than mild  **Preoperative coronary angiography**  Left main disease  3-vessel disease  2-vessel disease  1-vessel disease | 1.54 ± 6.93  3.86 ± 11.90  12.53 ± 1.89  6.77 ± 1.90  1.42 ± 1.65  180 (39.82%)  150.83 ± 40.23  93.87 ± 36.91  41.75 ± 10.80  0.72 ± 1.80  57.49 ± 12.31  351 (77.65%)  53 (11.73%)  48 (10.62%)  11 (2.43%)  2 (0.44%)  1 (0.22%)  99 (21.90%)  355 (78.54%)  95 (21.02%)  2 (0.44%) | 1.84 ± 9.34  4.91 ± 20.39  12.69 ± 1.83  7.01 ± 1.84  1.43 ± 1.58  206 (45.58%)  153.44 ± 41.76  93.72 ± 35.82  41.84 ± 11.06  0.64 ± 1.50  57.11 ± 12.21  353 (78.10%)  49 (10.84%)  50 (11.06%)  13 (2.88%)  2 (0.44%)  2 (0.44%)  95 (21.02%)  356 (78.76%)  93 (20.58%)  3 (0.66%) | | 0.036  0.063  0.084  0.126  0.005  0.116  0.064  0.004  0.008  0.046  0.031  0.030  0.028  <0.001  0.038  0.022  0.005  0.011  0.030 | |
| **Operative characteristics**  Type of surgery  Elective  Urgent  Number of anastomoses  Graft  Left internal thoracic artery  In situ graft  Composite graft  Free graft  Right internal thoracic artery  In situ graft  Composite graft  1  2  Free graft  Medications  Aspirin  P2Y12 inhibitors^*^  Beta-blockers  ACE inhibitors or ARB  Statins | 447 (98.89%)  5 (1.11%)  4.17 ± 0.97  439 (97.12%)  18 (3.98%)  6 (1.33%)  8 (1.77%)  415 (91.81%)  18 (3.98%)  15 (3.32%)  447 (98.9%)  304 (67.3%)  364 (80.5%)  127 (28.1%)  351 (77.7%) | 445 (98.45%)  7 (1.55%)  4.15 ± 1.00  441 (97.57%)  22 (4.87%)  6 (1.33%)  6 (1.33%)  416 (92.04%)  23 (5.09%)  13 (2.88%)  449 (99.3%)  329 (72.8%)  363 (80.3%)  129 (28.5%)  371 (82.1%) | | 0.039  0.022  0.028  0.043  <0.001  0.036  0.088  0.026  0.047  0.121  0.010  0.006  0.111 | |

Data are expressed as numbers (%) or mean ± SD values.

^*^P2Y12 inhibitors included clopidogrel, ticagrelor, and prasugrel.

*DM* diabetes mellitus, *SMD* standardized mean difference, *NYHA* New York Heart Association, *CCS* Canadian Cardiovascular Society Angina Score, *CK-MB* Creatine kinase-MB, *GFR* glomerular filtration rate, *MR* mitral regurgitation, *AR* aortic regurgitation,*TR* tricuspid regurgitation, *ACE* angiotensin-converting enzyme, *ARB* angiotensin receptor blocker
