# Supplementary material for: Impact of diabetes mellitus on long-term clinical and graft outcomes after off-pump coronary artery bypass grafting with pure bilateral skeletonized internal thoracic artery grafts
Source: Cardiovasc Diabetol. 2022 Nov 15;21:243. doi: 10.1186/s12933-022-01687-2 (PMC9667562; doi:10.1186/s12933-022-01687-2)
Supplement: Supplementary file 2 — Additional file 2: Figure S1. Love plots for propensity score matching (A) between DM and non-DM groups and (B) between well-controlled and poorly controlled DM groups. Figure S2. Cumulative incidence curves for cardiac death (A), myocardial infarction (B), repeat revascularization (C), and MACE (D) according to preoperative HbA1c. Non-cardiac death was accounted as a competing event in the Fine-Gray model. Figure S3. Cumulative incidence curves for graft failure (A) and stroke (B) according to preoperative HbA1c. Non-cardiac death was accounted as a competing event in the Fine-Gray model. [file 12933_2022_1687_MOESM2_ESM.docx]

**
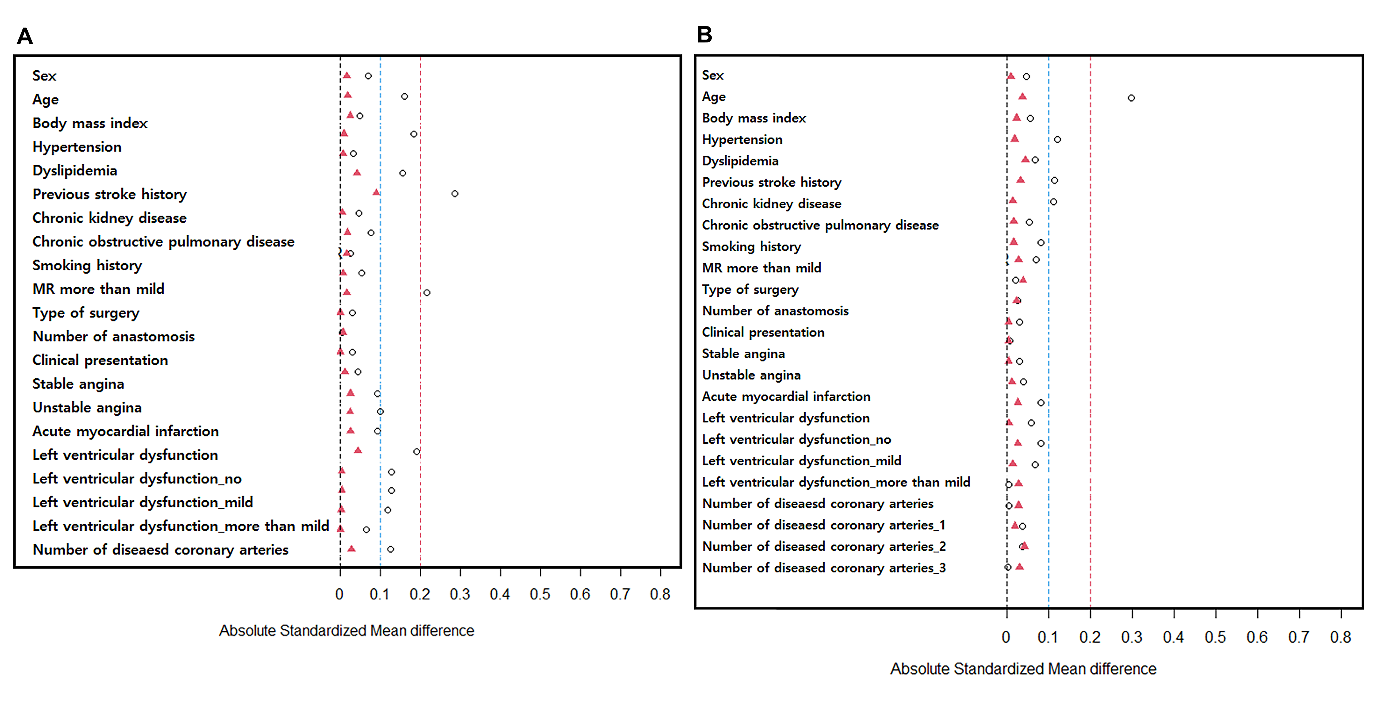
**

**Figure S1.** Love plots for propensity score matching (A) between DM and non-DM groups and (B) between well-controlled and poorly controlled DM groups**.**

DM, diabetes mellitus.


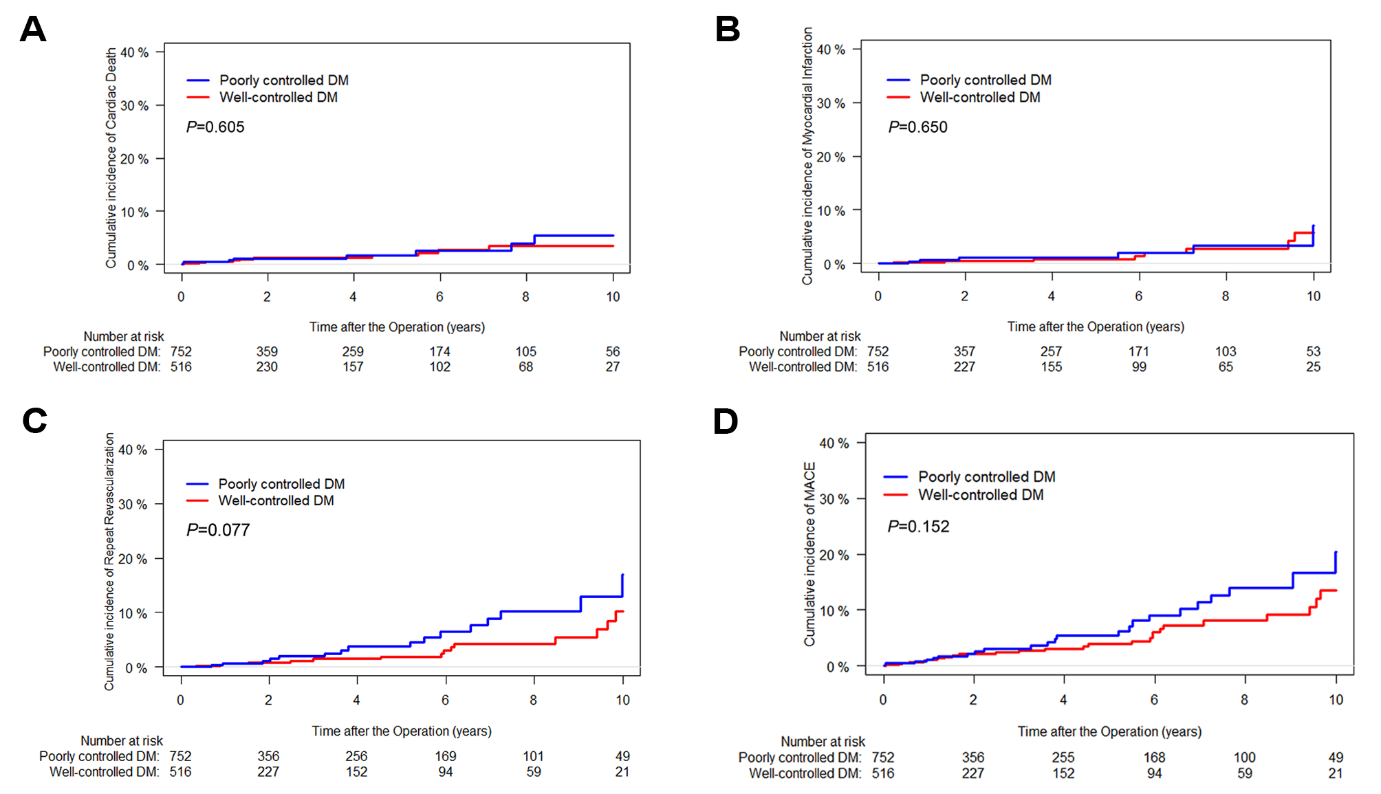


**Figure S2.** Cumulative incidence curves for cardiac death (A), myocardial infarction (B), repeat revascularization (C), and MACE (D) according to preoperative HbA1c. Non-cardiac death was accounted as a competing event in the Fine-Gray model.

MACE, major adverse cardiovascular and cerebrovascular events; HbA1c, hemoglobin A1c.


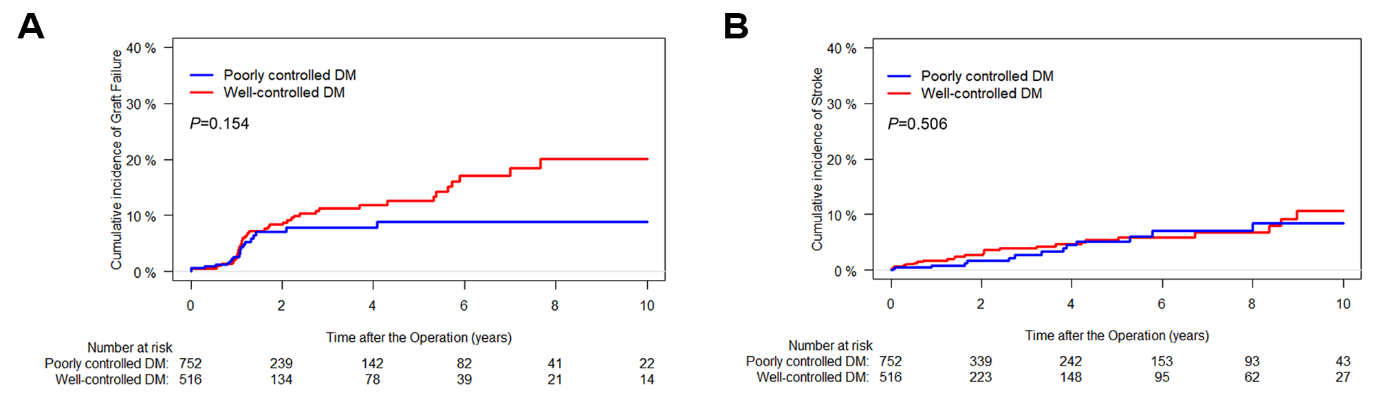


**Figure S3.** Cumulative incidence curves for graft failure (A) and stroke (B) according to preoperative HbA1c. Non-cardiac death was accounted as a competing event in the Fine-Gray model.

HbA1c, hemoglobin A1c.
